# Precision in harsh environments

**DOI:** 10.1038/micronano.2016.48

**Published:** 2016-10-10

**Authors:** Paddy French, Gijs Krijnen, Fred Roozeboom

**Affiliations:** 1 Faculty of Electrical Engineering, Mathematics and Computer Science, TU Delft, Delft, The Netherlands; 2 Faculty of Electrical Engineering, Mathematics and Computer Science, Twente University, Enschede, The Netherlands; 3 Eindhoven University of Technology, Department of Applied Physics, PO Box 513 5600 MB, Eindhoven, The Netherlands; 4 TNO Holst Centre, High Tech Campus 21, 5656 AE, Eindhoven, The Netherlands

**Keywords:** atomic layer deposition, harsh environments, micro/nano-fabrication technology, packaging, sensors

## Abstract

Microsystems are increasingly being applied in harsh and/or inaccessible environments, but many markets expect the same level of functionality for long periods of time. Harsh environments cover areas that can be subjected to high temperature, (bio)-chemical and mechanical disturbances, electromagnetic noise, radiation, or high vacuum. In the field of actuators, the devices must maintain stringent accuracy specifications for displacement, force, and response times, among others. These new requirements present additional challenges in the compensation for or elimination of cross-sensitivities. Many state-of-the-art precision devices lose their precision and reliability when exposed to harsh environments. It is also important that advanced sensor and actuator systems maintain maximum autonomy such that the devices can operate independently with low maintenance. The next-generation microsystems will be deployed in remote and/or inaccessible and harsh environments that present many challenges to sensor design, materials, device functionality, and packaging. All of these aspects of integrated sensors and actuator microsystems require a multidisciplinary approach to overcome these challenges. The main areas of importance are in the fields of materials science, micro/nano-fabrication technology, device design, circuitry and systems, (first-level) packaging, and measurement strategy. This study examines the challenges presented by harsh environments and investigates the required approaches. Examples of successful devices are also given.

## Introduction

The earliest known weights and measures systems date to approximately the 4th or 3rd millennium BC and was developed among the ancient peoples of Egypt, Mesopotamia, the Indus Valley, and perhaps Elam (Iran). Over the centuries, different weights and measures systems have emerged. Sensors were designed that could use these systems to measure and define a wide range of parameters. With the industrial revolution, the need for measurements increased greatly, and measurement devices were increasingly used in harsh environments. In the early days, sensors were quite simple and relied on the operator to control the machinery. With the advent of automation, it became increasingly necessary to use sophisticated sensors as components of a control system. These sensors were designed to simply control the machinery. In the automotive industry, this approach was used to improve performance. In recent years, an increasing need has arisen for sensors for environmental control, for example, regulation of the exhaust from automobiles or from the process industry. The source of the harshness might not always be the measurand itself, for example, in the case of measuring pressure at high temperature.

This study defines a harsh environment as any environment that can impede the operation of the device. Harshness can originate from various sources, and examples include the following:

- High pressure
- High temperature
- Shock/high vibration
- Radiation
- Harsh chemicals
- Humidity
- Biological (including inside the body for medical implants).

Many industries must address these environments, and selected examples are given in Table 1. The extent of the harshness and the limitations on design differ from application to application.

The approach chosen depends on the domain of the harshness and the application. Some examples are given in Table 2.

Table 2 shows a matrix of the harsh conditions (columns) and hierarchical levels at which the specific harsh conditions can be counteracted (rows). The number of + signs indicates the appropriateness of the given level for improved resistance against harshness. For example, chemical harsh environments can best be counteracted by proper material choices and packaging as well as the choice of fabrication technology. Typical approaches to improving resistance to harsh environments are listed below:

- Materials
  ○ Chemically inert
  ○ High glass or melting temperature
  ○ Dense materials to reduce device exposure to radiation
  ○ Material combinations and alloying
  ○ Fabrication method, conditions, annealing
  ○ Additional layers (for example, to prevent delamination and increase resilience), additives
  ○ Special zones to absorb mechanical/chemical loading or thermal cycling
  ○ Choice of measurand (for example, a derivative quantity)
  ○ Special zones to absorb mechanical/chemical loading or thermal cycling
  ○ Materials used in packaging (for example, chemically inert)
  ○ Isolation of the electronics from the harsh environment (for example, high temperature)
  ○ Modes of operation (for example, intermittent to prevent self-heating or low power)
- Technology
  ○ Fabrication method, conditions, annealing
  ○ Additional layers (for example, to prevent delamination and increase resilience), additives
  ○ Special zones to absorb mechanical/chemical loading or thermal cycling
  ○ Choice of measurand (for example, a derivative quantity)
  ○ Special zones to absorb mechanical/chemical loading or thermal cycling
  ○ Materials used in packaging (for example, chemically inert)
  ○ Isolation of the electronics from the harsh environment (for example, high temperature)
  ○ Modes of operation (for example, intermittent to prevent self-heating or low power)
- Device design
  ○ Special zones to absorb mechanical/chemical loading or thermal cycling
  ○ Choice of measurand (for example, a derivative quantity)
  ○ Special zones to absorb mechanical/chemical loading or thermal cycling
  ○ Materials used in packaging (for example, chemically inert)
  ○ Isolation of the electronics from the harsh environment (for example, high temperature)
  ○ Modes of operation (for example, intermittent to prevent self-heating or low power)
- Packaging
  ○ Special zones to absorb mechanical/chemical loading or thermal cycling
  ○ Materials used in packaging (for example, chemically inert)
  ○ Isolation of the electronics from the harsh environment (for example, high temperature)
  ○ Modes of operation (for example, intermittent to prevent self-heating or low power)
- System
  ○ Isolation of the electronics from the harsh environment (for example, high temperature)
  ○ Modes of operation (for example, intermittent to prevent self-heating or low power)

As an example, in certain cases, the best approach might be optical (that is, often non-invasive) sensing. This method can allow remote sensing in applications where the sensing system is not required to be exposed to the harsh environment, for example, excitation by a laser and measurement of the optical absorption or scattering in the area of interest^1^ or use of fiber materials that can withstand high temperatures, such as sapphire^2^. Optical signals in fibers are also not affected by electrical noise^3^. Coating layers, such as those used with atomic layer deposition (ALD)^4,5^, may protect the device from harsh chemical environments as well as the use of chemically inert materials such as SiC^6,7^. For high-temperature applications, wide band gap materials such as SiC, GaN, AlN or diamond^8–10^ may be used. More recently, interest in the use of graphene has increased for high temperature applications^11^.

The following sections examine the sources of harshness and the approaches required to address them and allow accurate measurement.

## Processing and materials

### Substrate materials

Standard electronics are fabricated using single-crystalline silicon, which is an excellent material from both electrical and mechanical perspectives. However, silicon encounters limitations with respect to temperature range. Silicon-based electronic devices are not usually operated above 150 °C owing to junction leakage. Above ~200 °C, the material becomes intrinsic owing to thermal generation of electron–hole pairs. This effect can be offset locally using the exclusion principle^12^. An alternative approach is to use a semiconductor with a wider band gap, such as SiC, GaN, and AlN. However, the Integrated Circuit (IC) technology for these materials is less well developed, and the substrates are more expensive.

### Thin-film deposition

Modern semiconductor thin-film deposition techniques are treated in detail in textbooks^13^. A non-exhaustive list of current thin-film deposition methods is shown in Table 3 together with short descriptions.

General selection criteria for thin-film deposition are given in Table 4.

### Brief comparison of the most popular thin-film technologies

For over 50 years, the most popular thin-film deposition methods in industry have been physical vapour deposition (PVD) and chemical vapour deposition (CVD), with atomic layer deposition (ALD) emerging in applications where nanometer-size layer thicknesses or pinhole free layers are crucial. In particular, spatial ALD has potential as an interesting newcomer^14,15^. On the basis of the selection criteria listed above, one can compare the different deposition methods compiled in Table 5.

#### CVD

This method is widely used in the deposition of thin films and is available in three forms: atmospheric-pressure CVD (APCVD), low-pressure CVD (LPCVD), and plasma-enhanced CVD (PECVD). The main layers deposited are silicon oxide, silicon nitride, silicon and silicon carbide (SiC). For harsh environments, SiC is of particular interest because it is chemically inert and can operate at high temperatures. Using PECVD, SiC can be deposited at temperatures <400 °C, thus making it suitable as a coating layer for an IC intended for use in harsh chemical environments. This layer has been observed to be pinhole-free for thicknesses greater than ~200 nm and can be used as the mechanical material for pressure sensors^16^. LPCVD SiC can also be used, but this material has a deposition temperature above 700 °C and thus cannot be used as a coating layer for standard circuitry (unless an alternative metal layer is used). Other wide band gap semiconductors such as GaN and AlN are also useful materials for high temperature applications.

#### ALD

This technique is used to deposit films in an atomic layer-by-layer manner^4,5^, which enables the deposition of notably thin pinhole-free layers of high quality. ALD was first published under the name molecular layering in the early 1960s by Professor Kol’tsov from Leningrad Technological Institute, although the basic concept was proposed by Professor Aleskovski in his PhD thesis in 1952. The characteristic feature that distinguishes ALD from other deposition technologies such as CVD, PVD, sol–gel synthesis, and spray pyrolysis is the self-limiting chemisorption of precursors in each half-cycle^5^. This feature makes ALD unique in sub-nanometer film thickness and conformality control, offering next to (nearly) equal growth-per-cycle values for identical precursors in different equipment^17^. The remaining drawback of conventional ALD that prevents it from cost-effective commercial use is the low deposition rate, but this shortcoming has been largely overcome by the launch of spatial atmospheric ALD^5^.

Selected examples of ALD processes and applications are given in Table 6, which is based on Refs. 4,5,15,18–23.

The high quality of the layer makes it highly suitable for biomedical applications^4^ and for the use in harsh chemical environments in which the underlying layers must be protected ^24,25^.

#### Graphene

This material is an *sp*^2^-hybridized allotrope of carbon in the form of a two-dimensional, atomic-scale, hexagonal lattice in which one atom forms each vertex. The material is 200 times stronger than steel by weight and has high thermal and electrical conductance. Graphene has a tensile strength of 130 GPa and a Young’s modulus of 1 TPa, is able to operate at high temperature, is suitable for harsh chemical environments and is acceptable as a coating layer in medical implants^26^.

#### Polymers

Polymers are composed of a large, chain-like molecular structure made of monomers, which are covalently linked in three-dimensional networks. There are many examples of both natural and synthetic polymers and polymers have found numerous applications in the sensor field. One of these applications is in medicine where a number of polymers have been found to be suitable for both *in vitro* and *in vivo* devices. These polymers do not generate any bodily reactions and are also able to withstand the biological environment. Examples of these polymers include the following:

- Natural
  ○ Plants: cellulose, natural rubber
  ○ Animals: collagen, heparin, DNA
  ○ Parylene
  ○ Silicone rubber
  ○ Polyethylene
  ○ Polypropylene
  ○ Polymethyl methacrylate (PMMA)
  ○ Polyvinyl chloride
  ○ Polyether ether ketone (PEEK)
- Synthetic
  ○ Parylene
  ○ Silicone rubber
  ○ Polyethylene
  ○ Polypropylene
  ○ Polymethyl methacrylate (PMMA)
  ○ Polyvinyl chloride
  ○ Polyether ether ketone (PEEK)

Parylene is a trade name for a variety of chemical vapor-deposited poly(*p*-xylylene) polymers used as moisture and dielectric barriers. The most common of these materials is parylene-C, which was first discovered by Szwarc in 1947 and is suitable for a range of applications, including medical implants^27–32^. Parylene is deposited by selecting a raw dimer of the material and heating it to nearly 150 °C. The vapor is pulled out under vacuum and heated to high temperature, which allows for sublimation and splitting of the molecule into a monomer. This monomer is subsequently further drawn by vacuum for deposition onto the required substrate.

Silicone rubber is well known for its elastic behavior with a maximum elongation that can range from 90 to 900% depending on the composition^33^. This material can form watertight seals and is water-repellent, although rather permeable to gas, which is an interesting combination for medical applications. Combination with liquid metals allows for use in strain sensing with up to 50% change in resistance and application, for example, in smart gloves^34,35^ and soft artificial skin^36^.

PMMA is probably better known under the name ‘plexiglas’ and is a low-cost, optically transparent material that has numerous applications in sensing. PMMA is often used in the formation of microfluidic chips^37^, for example, in the form of molded structures.

PEEK is a semi-crystalline plastic used abundantly in engineering components owing to its mechanical strength, high wear resistance, chemical inertness in organic, and aqueous environments, high maximum operating temperature (up to 250 °C) and low thermal degradation. Although PEEK has relatively high glass transition and melting temperatures, it can be processed by molding and extrusion, computer numeric controlled machining^38^, and recently even through additive manufacturing by either fused deposition modeling (FDM)^39^ or selective laser sintering^40^. The properties of PEEK make it a biocompatible material that is used in the reconstruction of large cranial osseous defects^41^, among other applications.

#### Ceramics

Ceramics span a wide range of inorganic materials that can be highly ordered or amorphous (for example, glass). Ceramics such as sintered alumina are thermally and chemically resistant and can withstand temperatures as high as >2000 °C (Ref. 42) and aggressive applications in, for example, oven linings and plasma-etching equipment^43^. Some ceramics are also piezoelectrics and can therefore be applied in both sensors and microactuators. A review of the development of piezoelectric ceramics can be found in Ref. 44. In terms of medical applications, ceramics have been used in dental and orthopedic implants. For these applications, ceramics are robust and biocompatible (that is, they do not generate any reactions from the body). Ceramics have replaced many metal devices for implants^45^, and examples exist of biodegradable ceramics for bone implants^46^. Glass is used in the encapsulation of implants.

## Harsh environments

This section discusses the different types of harsh environments. In certain cases, attempts are made to measure parameters such as high temperature or alternative parameters at high temperature. There are many such examples within different signal domains.

### High temperature

Silicon is an excellent material for sensors but has certain limitations in terms of its process temperature window (the temperature tolerance range in which device fabrication can take place) and device-operating temperature range.

**Figure 1:**
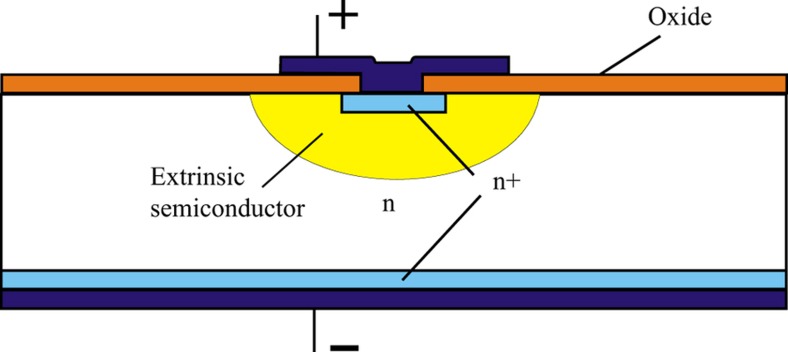
Basic structure for use of the exclusion principle.

If the device uses a p–n junction, the temperature is limited to ~125 °C. Above this temperature, the junction suffers from leakage. Near 200 °C, extrinsic silicon is reverted to intrinsic silicon owing to the high generation of electron–hole pairs. This transition can be extended to higher temperatures for small volumes of silicon (which might contain the device) using the exclusion principle^12^. The device is designed and biased such that a small contact must supply a large number of minority carriers, which means that if the semiconductor begins to generate large numbers of electron-hole pairs, the minority carriers are immediately absorbed and the volume remains an extrinsic semiconductor. If we are able to fabricate devices within this region, they can continue to operate normally at temperatures above the transition. In this work^12^, Hall devices were fabricated to operate at up to 500 °C. The basic principle is shown in Figure 1.

For direct temperature measurements, platinum resistors can be a good option. Platinum is stable up to ~1000^ ^°C (Ref. 47), and platinum-based thermocouples are widely used in temperature measurement. The materials used in the construction determine the device sensitivity and also the temperature range. Certain thermocouples are able to measure temperatures up to 3000 °C (Ref. 48). These high-temperature measurements require refractory (that is, high melting point) materials such as tungsten and rhenium.

**Figure 2:**
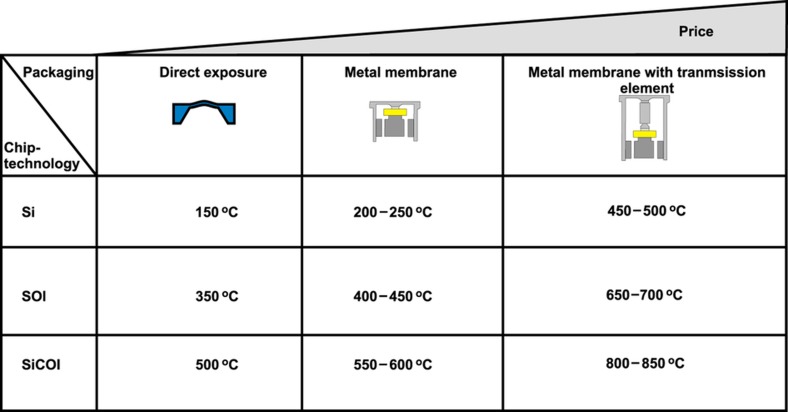
Different approaches to high temperature/pressure sensing. Adapted from Ref. 55.

In applications such as pressure sensing at high temperature, the sensor must be exposed to the environment. One option is to use wide band gap semiconductor materials that maintain their electrical properties up to higher temperatures. For example, SiC power devices have been operated successfully at temperatures of ~600 °C. Diamond devices have been found to operate at even higher temperatures, and Schottky devices have operated at 1000 °C (Ref. 49). Ceramic materials and metals such as tungsten maintain their mechanical properties at notably high temperatures (>1000 °C). These alternative materials can be combined with device/package designs that protect the sensing element from the high environmental temperatures. This approach is illustrated in Figure 2. The use of a silicon membrane in direct contact with the environment limits the working temperature for measuring pressure. If p–n junctions are used, the temperature is limited to below 150 °C. Above 500 °C, the good mechanical properties are lost, and the materials become plastically deformable^50^. The temperature range can be extended using a metal membrane with a mechanical connection to the chip. In this case, the pressure can be transduced to a pressure sensor via an incompressible fluid or through a hard contact to a stress sensor. Using the same basic principle, the sensor chip can be further isolated from the environment by adding a longer mechanical element that allows for further thermal isolation, as shown in the top right option, which greatly improves the temperature range, although it also increases the costs.

The use of SOI wafers^51–53^ or polysilicon piezoresistors^54^ can also increase the temperature range because these materials do not rely on p–n junctions. Further improvement can be achieved by selecting a wider bandgap material such as SiC or SiC on an insulator. All of these semiconductors have limitations in terms of their temperature range and bursting pressure. If a metal or other material is used as the membrane (Figure 2; Ref. 55), the material and thickness of the membrane can be chosen to meet the pressure and temperature requirements.

An alternative approach to high temperature application is the use of optical fibers. In the oil industry, for example, in the drilling of holes and pipes, long distances often lie between the point of measurement, and the readout in addition to high temperature and high electrical noise in the environment. In this case, optical fibers offer the best option and fibers capable of withstanding high temperatures can be used^56^. Alternatively, free-space optics can be used^57^. In this case, the light source and the receiver are isolated by distance from the harsh environment, and only the light passes through the device.

**Figure 3:**
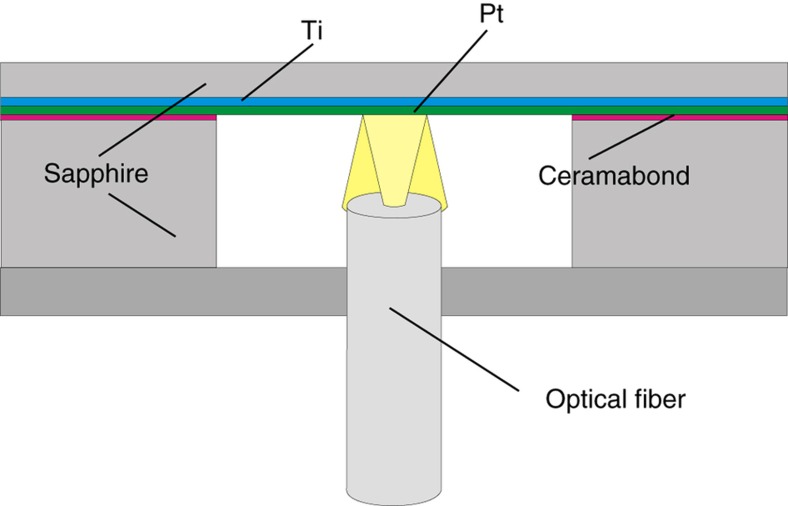
Optical fiber pressure sensor. On the basis of Ref. 60.

With fiber-optic devices, the temperature sensitive components of the system can be sheltered from high ambient temperatures. The measurement process can use interference^58^ or Fabry–Pérot interferometry^59^. An example of an optical-fiber pressure sensor is given in Figure 3 (Ref. 60).

**Figure 4:**
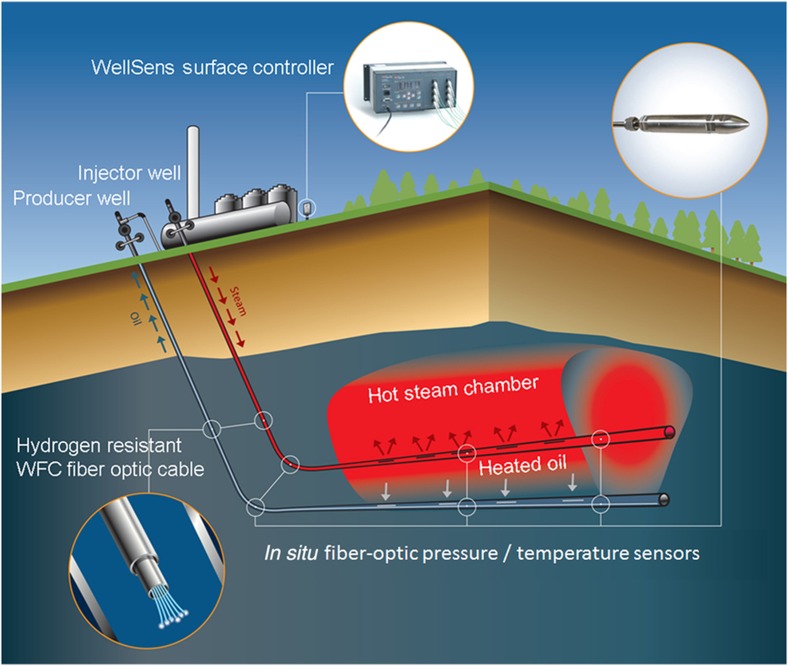
*In situ* monitoring of a steam-assisted gravity drainage (SAGD) well with unique white light Fabry–Pérot interferometer fiber-optic pressure and temperature sensors constructed from highly durable and corrosion-resistant sapphire material. Courtesy of Opsens Solutions Inc., Quebec, Canada.

In extraction and recovery in the oil industry, the point of measurement might be located >1 km from the instrumentation. Furthermore, the point of measurement and the path to the instrumentation may be at high temperature and also may be subjected to high electrical interference. An optical system can address many of these problems, and one such example is given in Figure 4. The use of optical techniques offers many opportunities for operation at high temperatures^61^.

The operating temperature range of standard CMOS can be increased by careful consideration of the design^62,63^. Through careful design of the preamplifier, the system was able to operate at temperatures up to 275 °C.

### Low temperature

Extremely low temperatures (<100 K) also create challenges for sensors and the electronics. Fibre Bragg gratings have been shown to work effectively at extremely low temperatures^64^, as have a number of other devices^65,66^. At moderately low temperatures, approximately −50 °C, problems occur with the packaging and the batteries in devices. As the temperature decreases further to cryogenic temperatures and below, the challenges greatly increase. Silicon continues to operate, but adjustments to the processing and the design are required. An overview of the issues for operation at cryogenic temperatures and below is given in Ref. 67. Enhanced operation at these low temperatures using high-K dielectrics can aid in maintaining correct operation^68^.

### High pressure

Silicon membrane pressure sensors are excellent for low to medium pressure (0–300 bar)^69^, but might easily fail when overloaded due the brittleness of silicon. However, in many industries, such as the automotive and process industries, the pressure to be measured might lie in the range of 2000–3000 bar. For these higher pressures, alternative membrane material can be used. The main approach uses a metal membrane that determines the pressure range and transfers the value to the sensing element via a fluid or fixed connection. This method is similar to the approach used in sensors that operate at high temperature. One example deposits polysilicon onto a stainless steel substrate^70^, although this method generates additional processing challenges. The best option is to use sputtered oxide as an insulator because it can be formed on a wide range of substrates at relatively low temperature. The type of stainless steel used was suitable for temperatures up to ~500 °C. Polysilicon piezoresistors were deposited at low temperature and laser-annealed to avoid excessive heating of the substrate. High pressure/high temperature measurement systems have also been developed using optical techniques^71^, and ceramics can be applied for this purpose^72^.

### Shock/high acceleration

In some military applications, measurement devices are expected to survive high acceleration and shock during operation. Accelerometers designed for low acceleration still must withstand the high shock/high acceleration, which can be achieved using stoppers that prevent the mass from moving too far. Selected accelerometers use oil in the cavity, which prevents the device from oscillating at resonant frequencies and protects it from shock^73^.

### Radiation

**Figure 5:**
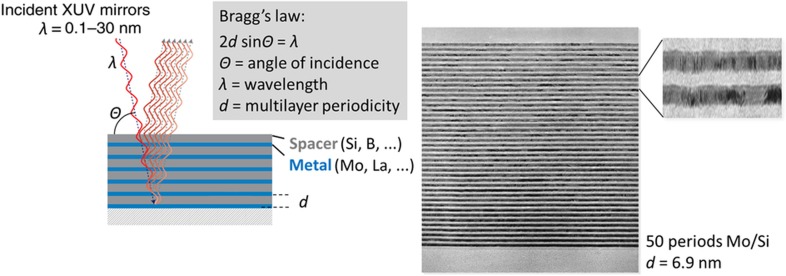
Principle and cross-sectional TEM images of a multi-layer UV mirror currently grown by e-beam evaporation and ion beam sputter deposition^75,76^. Courtesy: F. Bijkerk. TEM, transmission electron microscopy; UV, ultraviolet.

Measurement of radiation or operation in an environment with radiation presents many challenges, depending on the type of radiation. In space applications, devices might be exposed to high levels of ultraviolet (UV) radiation from the sun. Certain systems can be protected from this radiation via installation in the satellite or spacecraft, but others must be exposed to be able to measure. In satellite applications, the devices might also be exposed to sharp swings in temperature. In medical applications, the devices could be exposed to X-ray or proton bombardment. Modern lithography uses extreme UV, which is reflected by at least eight Bragg reflector mirrors that compose the reticule stage and the wafer stage in the optics of an EUV system. Still, the current mirror reflectivity record is still only 70.3% of the EUV light. Current state-of-the-art mirrors are produced by e-beam evaporation and ion beam sputter deposition of superlattices (Mo/Si; see Figure 5 (Ref. 74)) with B_4_C interlayer diffusion barriers to improve thermal diffusion resistance^75,76^. These devices yield >70% EUV reflectivity, although 75% yield is theoretically possible with superior mirror surface protection and new absorber and spacer material layers. In addition to the ultrathin layer deposition techniques that are currently used in EUV mirror manufacturing (for example, e-beam evaporation and ion beam sputter deposition), atomic layer deposition can be developed to further improve the so-called spectral purity of the multilayer stacks. Examples are found in anti-reflection coatings designed to improve DUV contrast for inspection (Al_2_O_3_, SiON), absorber layers to absorb EUV for image formation (for example, Ta(B)N, TaSi(N)), and buffer/capping layers to protect the mirror stack from absorber-etch damage and from unexpected oxidation, and blistering due to hydrogen radical diffusion into the mirror layer stack, for example, Ru, TiO_2_, Nb, and B_4_C (Ref. 77). Efforts have also been put forth to increase the emissivity (currently ~30%) of the layer stack to counteract the heating by IR and DUV radiation from the EUV source.

**Figure 6:**
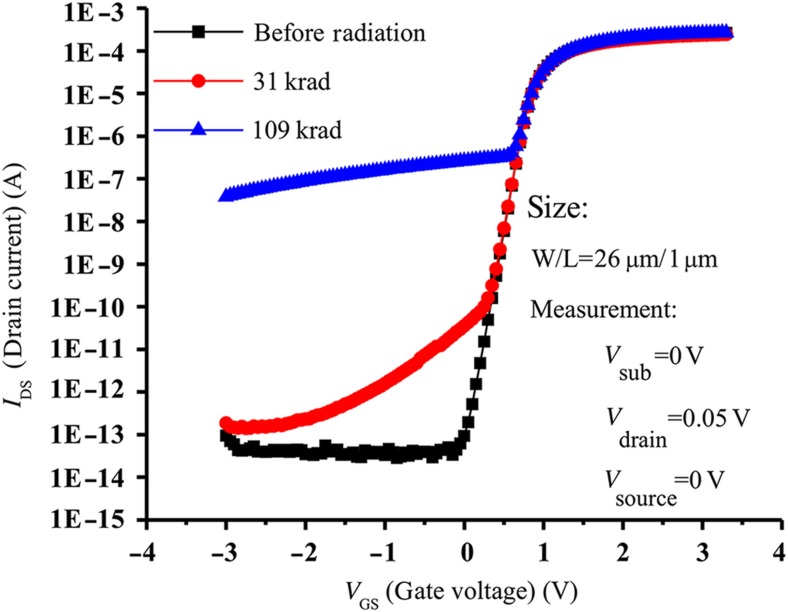
Effects of X-ray radiation on MOS transistor characteristics^78^. MOS, metal–oxide–semiconductor.

**Figure 7:**
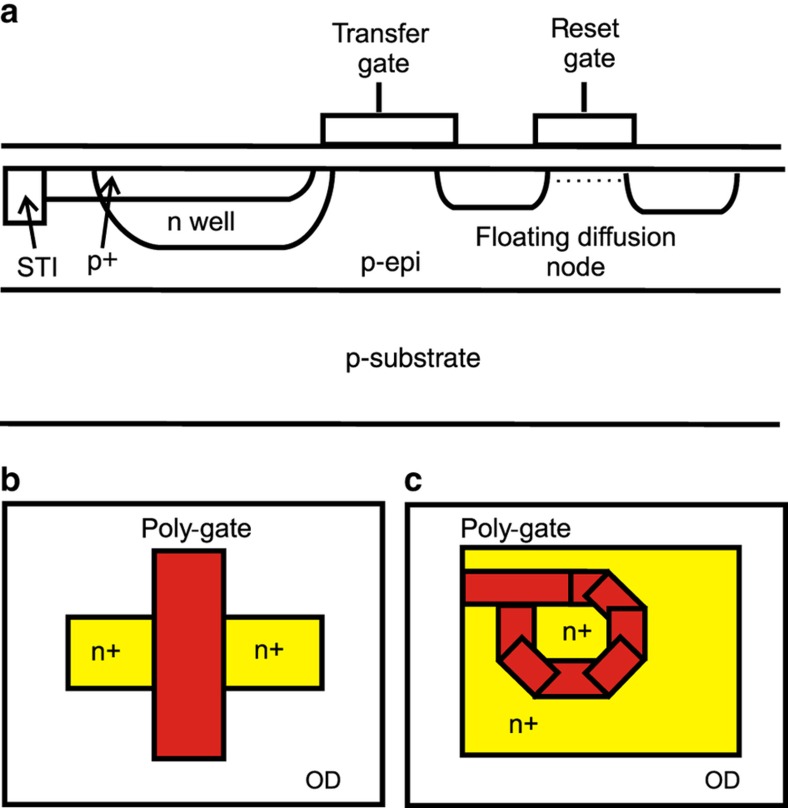
(**a**) Cross-section of in-pixel elementary devices, (**b**) regular layout of a MOSFET, (**c**) enclosed layout of a MOSFET^78^. MOSFET, metal–oxide–semiconductor field-effect transistor.

**Figure 8:**
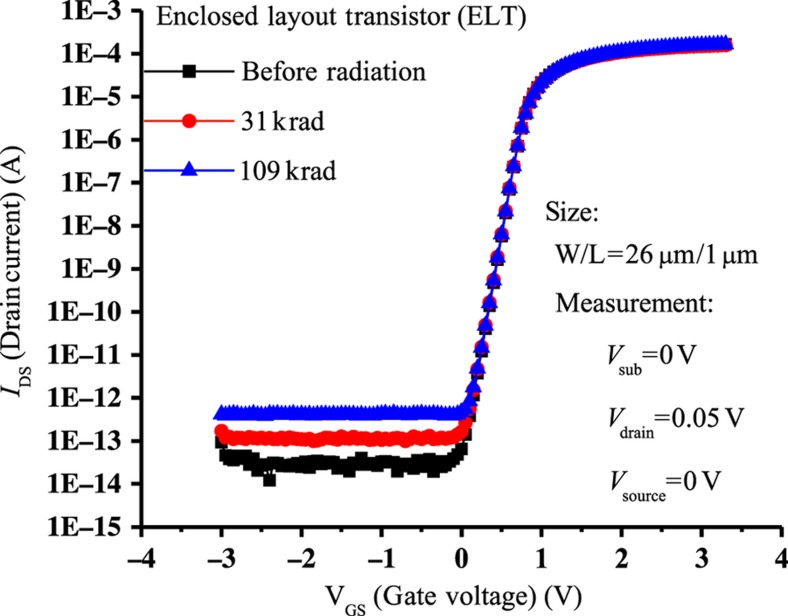
Reduced radiation effects using the enclosed layout transistor^78^.

X-ray radiation can have notably harmful effects on operation of MOS devices, and exposure to X-rays can shift the transistor characteristics^78^. The shift with increasing exposure is shown in Figure 6 (Ref. 78). This effect can be greatly reduced through layout design using an enclosed layout (Figure 7 (Ref. 78)) with the resulting improvement in radiation hardness shown in Figure 8 (Ref. 79).

**Figure 9:**
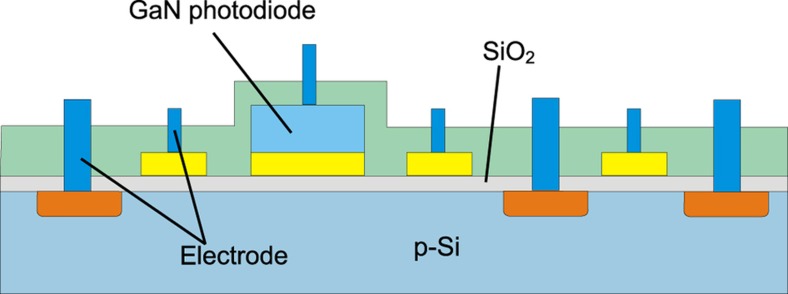
Cross-section of a UV sensor that uses a GaN diode. Adapted from Ref. 79. UV, ultraviolet.

Alternative materials can be used to measure radiation signals outside the normal range for silicon. One such approach uses GaN photodiodes formed on top of a standard IC. This approach enables UV measurement, and readout can be accomplished using standard IC technology. A cross-section of this device is shown in Figure 9 (Ref. 79).

### Harsh chemical environment

**Figure 10:**
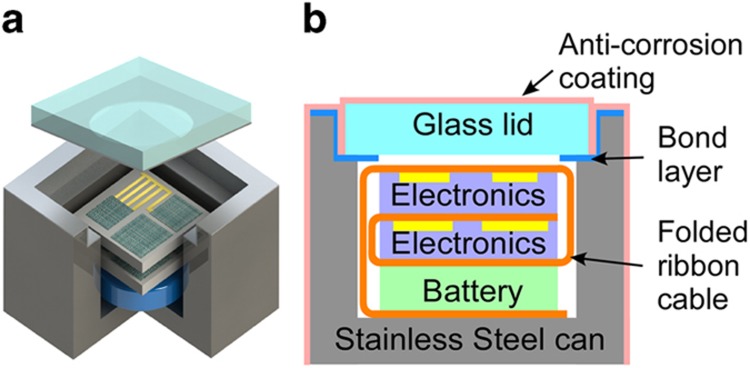
Package designed for high-pressure applications in a saline environment^83^. Reproduced with kind permission from Y Gianchandani and Tao Li.

Aggressive chemicals affect not only the measurement layer but also the packaging. It is therefore essential that all aspects of the device are considered during development. In certain cases, a layer is used to react with the target chemical to be measured. Many different types of sensors are available for chemical measurement, including electrochemical, chemFET, and optical types. The chemFET option is one of a group of devices that also include devices for measuring ions (ISFET) and enzymes (ENFET). These devices are based on standard MOS transistors in which the gate has been modified to enable the measurement^80^. Because this device is based on silicon technology, it has a limited temperature range. Tobias *et al*^81^ reported useful properties at the interface between oxide and SiC at temperatures greater than 700 °C (Ref. 81). A review of MOS-based hydrogen sensors using SiC technology can be found in Ref. 82. In certain cases, the aggressiveness of the chemical requires a protection layer or a measurement layer that can withstand the environment. Such a solution is depicted in Figure 10 (Ref. 83).

**Figure 11:**
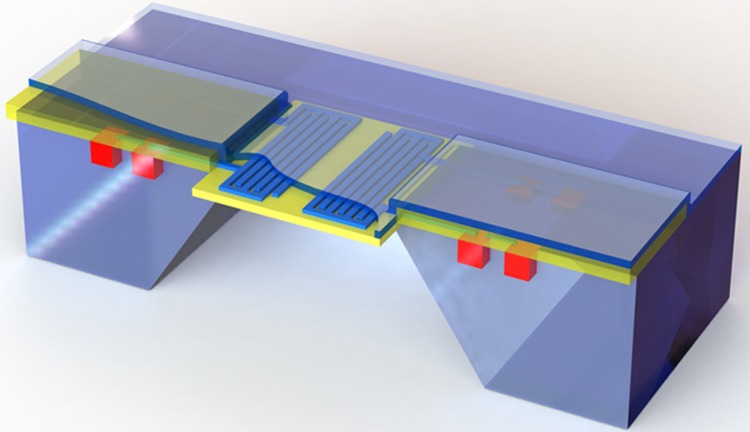
Capacitive ammonia sensor using porous SiC as the sensing layer and SiC as a protection layer for the electronics.

Silicon carbide is an excellent candidate for harsh chemical environments because it is chemically inert and can also operate at high temperatures. In its porous form, it can be used as an ammonia sensor. Ammonia is highly aggressive and can destroy both many sensing materials, such as silicon, and commonly used metals, such as aluminum. Porous silicon carbide can be used as a capacitive ammonia sensor with high reliability^84^. The basic structure is shown in Figure 11. The entire chip is covered with SiC as a protection layer, and only the area above the electrodes is rendered porous for sensing.

### Humidity

Humidity has always been an issue for ICs. Packaging must be hermitically sealed to ensure that humidity cannot reach the chip. Humidity results in corrosion of the metallization and even creates short circuits if it reaches under the passivation layer to the silicon itself. Humidity sensors must be exposed to this environment and therefore can suffer from reliability problems. Most humidity sensors are capacitive and use polymers or ceramics to absorb the water vapor and therefore change the capacitance^85–89^. With all of these devices, the remainder of the chip must be hermetically sealed to ensure reliable operation, but a further reliability issue remains with these devices. Both of these materials expand with absorbed moisture, which results in physical expansion. The continual expansion and contraction leads to stress on the adhesion between the sensing layer and the underlying substrate, which can lead to failure. One solution to this problem is to use porous silicon^90^. Porous silicon does not expand with moisture absorption, and therefore, the problem is resolved. However, if exposed to high humidity and elevated temperature, surface oxidation in the pores can lead to drift. A move to silicon carbide and use of a structure similar to that in Figure 11 might aid in solving the problem because SiC is far less reactive than silicon and therefore does not oxidize at these temperatures^91^.

### Medical implant/catheters

For medical implants, limitations exist on both sides. From the medical point of view, there are limitations on the materials that can be used and on the size and shape of the device to maintain biocompatibility, whereas from the device point of view, the implant must survive in a harsh environment. The materials that can be used in medical implants are limited by the constraints of biocompatibility. In many cases, coating layers are applied, such as polymers, parylene, PEEK (polyether ether ketone) or graphene^92–95^. A number of metals can also be used (depending on the location of the device), such as platinum, titanium, and TiN. An overview of the important issues with metals, ceramics, and polymers is given in Table 7 (Ref. 94).

The regulations depend upon the function of the device/material, where it is located, and for how long it remains in contact with tissue. The longer the contact with tissue, the tighter the regulations will be. Catheters, for example, will usually remain in the body for less than 72 h. Monitoring systems used after an operation might be in contact with tissue for days or perhaps a number of weeks. Long-term implants, such as cochlear implants and pacemakers, are typically implanted for 7–10 years. The FDA regulations depend on the location of the implant and the length of time that the device is in contact with living tissue. The three categories are <1 day, between 1 and 30 days, and >30 days. These regulations are covered under the standards ISO 10993-1 in the US^96^ and EN 30993-1 in Europe.

An overview of biomaterials can be found in Ref. 93, and the role of microsystems is described in Ref. 97. Many implanted devices require a wireless communication system with the outside environment^98^. In addition to the limitations on materials that can be used, the *in vivo* environment is highly aggressive, not only because of the chemical environment but also because the body attacks any foreign material. Catheters are generally in place for less than 72 h and maintain direct wired contact to the outside. The first patient treatment with cardiac catheters dates back to the 1950s with the work of Dotter. Silicon sensors were able to meet the space restrictions of this application and, with the correct coating/packaging, were able to withstand the environment and avoid generation of any unwanted reaction from the host. A pressure sensor that uses a membrane was a simple device that met all requirements and has been a successful commercial product for many years^99^. Companies such as NovaSensors (Buellton, CA, USA), Braun (Frankfurt, Germany), Edwards, Yilson Medical Technology, and Cook Medical (Bloomington, IN, USA) have pressure-monitoring catheters in the market, and many more companies are currently producing these types of devices. Most of the products are disposables and must therefore be manufactured at low cost. Furthermore, most commercial devices contain only a single sensor. However, use of silicon allows a number of sensors to be included on a single chip^100,101^. As stated above, catheters are generally used over periods of <72 h and maintain direct wired contact to the outside world, but implanted sensors often must remain in the body for longer periods and use wireless communication^102,103^. In addition, power for the devices must be considered. Batteries are one option, but inductive powering can be a better option^104^. Polymers are excellent materials for implants owing to the choice of biocompatible polymers because they are generally rather flexible and can also be used as a sensing layer. One such example is measurement of oxygen in tissue using optical techniques and a polymer in direct contact with the tissue^105^. Long-term implants include pacemakers^106^, deep brain probes^107^, prostheses^108^, cochlear implants^109^, and retinal implants^110^. Interest also exists in long-term implants for administration of insulin or other medicines for chronic conditions. In some cases, the sensor can be completely protected from the environment, but in other situations, the sensor must be exposed and therefore must withstand a harsh environment. For example, the cochlear implant operates in a saline solution for a number of years. The electrodes must be maintained as electrically active, which increases corrosion. Modern implants use plate platinum as the electrode. Future cochlear implants might be fabricated using IC technology, and in this case, TiN may be a better option for the electrodes^111,112^. In the field of medical implants, the challenges are broad and varied. In some applications, the combination of humidity, local pH, and body fluids can combine to erode both the electrode and the packaging.

## Testing and reliability

If any device is to achieve commercial success, the reliability of the devices must be demonstrated. Where possible, the testing should occur under more extreme conditions than in the intended application. Standard testing methods include high temperature/high humidity, temperature swings, radiation exposure, high salt solutions, and so on. These tests must be designed to best test the microsystem for the intended environment. At the European Space Agency, a wide range of test facilities is used to ensure that the devices can survive the environment^113^. In medical implants, biocompatibility tests are complicated and extensive^96^. In addition, the devices must be tested to maintain good functionality in the environment. Many tests are performed in salt solutions that are more concentrated than those encountered in applications. Reliability testing is an essential component of the development and must be adapted for each application. This requires a good understanding of the environment and the failure mechanisms^114^.

## Conclusions

A harsh environment is any environment that impedes the normal operation of a sensing or actuating device. A number of approaches can be used to address these problems, such as alternative materials, coating layers or design as well as protective device packaging. For each application, it is necessary to consider all of these aspects to successfully design and fabricate a reliable system.

## Figures and Tables

**Table 1:** Harsh environments in different industries

Industry	Harsh environment
Oil/process	Temperature, chemical, pressure
Automotive	Temperature, chemical, pressure, electric fields
Space	Temperature swings, radiation, high vacuum
Aircraft	Temperature, pressure, vibration, electric fields
Farming	Pesticides, chemical, biological
Medical implants	Biological (for example, stomach, colon, blood)

**Table 2:** Examples of harsh environments and domains that offer the most effective approaches

	Chemical	Thermal	Mechanical	EM loading*	Radiation
Materials	**++**	**++**	**+**		**+**
Technology	**+**	**++**	**+**		
Device design		**+**	**++**	**+**	
Packaging	**++**	**+**	**++**	**++**	**+**
System		**+**	**+**	**+**	**+**

* EM loading occurs when electromagnetic disturbances are present. EM: electromagnetic.

**Table 3:** Concise survey of mainstay thin-film deposition methods and characteristics

*Electroplating*
Film formation from chemicals in dissolved electrolytic solution placed onto substrate surface with a seed layer on top that is electrically biased.

*Spin coating*
Film formation from chemical reaction between liquid-phase sources (often sol–gel) that have been applied onto the rotating substrate and subsequently heated.

*Thermal oxidation*
Film formation by chemical oxidation of the substrate surface.

*Physical vapor deposition* (*PVD*)
Film formation by condensation of gasified source material directly transported from source to substrate through the gas phase via
Evaporation (thermal, E-beam)
Molecular beam epitaxy (MBE)
Pulsed laser deposition (PLD)
Reactive PVD
Sputtering (DC, DC magnetron, RF)

*Chemical vapor deposition* (*CVD*)
Film formation by chemical reaction between mixed gaseous source materials on a substrate surface using:
Atmospheric-pressure CVD (APCVD)
Low-pressure CVD (LPCVD)
Plasma-enhanced CVD (PECVD)
Metal-organic CVD (MOCVD)

*Atomic layer deposition* (*ALD*)
A sub-class of CVD with film formation via sequential cycling of self-limiting chemical half-reactions on the substrate surface. Each reaction cycle accounts for the deposition of a (sub)monolayer. The reaction can be activated by thermal energy or plasma enhancement or can be performed in the spatially divided regime^14^.
Thermal ALD
Plasma-enhanced ALD (PEALD)
Spatial ALD (S-ALD)

**Table 4:** Criteria for selection of thin-film techniques

Deposition rate
*Deposition directionality*
Directional: suitable for lift-off, 3D feature filling
Non-directional: suitable for step coverage

*Film thickness and composition uniformity*
Within-wafer
Wafer-to-wafer (single-wafer deposition) and run-to-run (batch deposition)

*Film quality* (*physical and chemical properties*)
Adhesion
Breakdown voltage
Film density, pinhole density
Grain size, grain boundary features, and grain orientation
Impurity level
Stoichiometry
Stress and yield strength
Hermeticity

*Functional materials to be deposited*
Metals/conductors
Dielectrics, magnetics, piezoelectrics, etc.
Polymers

Cost-of-ownership and operation cost

Abbreviation: 3D, three dimensional.

**Table 5:** Comparison of thin-film deposition methods

Process	Material	Substrate temperature (^o^C)	Deposition rate (Å s^−1^)	Directionality	Uniformity	Film density	Grain size (nm)	Impurity level	Cost
Thermal evaporation	Metal or low-melting point materials	50–100	1–20	Yes	Poor	Poor	10–100	High	Very low
E-beam evaporation	Both metal and dielectric	50–100	10–100	Yes	Poor	Poor	10–100	Low	High
Sputtering	Both metal and dielectric	–200	Metal –200 Dielectrics 1–10	Some degree	Very good	Good	–20	Low	High
PECVD	Mainly dielectrics	200–300	10–100	Some degree	Good	Good	10–100	Very low	Very high
LPCVD	Mainly dielectrics	600–1200	Metal –100 Dielectrics 1–10	Isotropic	Very good	Excellent	1–10	Very low	Very high
ALD (thermal)	Mainly dielectrics	50–300	0.1–1	Isotropic step conformal	Superior	Superior	1–10	Very low	Very high
ALD (plasma)	Mainly dielectrics	20–200	0.1–1	Isotropic	Superior	Superior	1–10	Very low	Very high
ALD (spatial)	Mainly dielectric	20–200	1–10	Isotropic step conformal	Superior	Superior	1–10	Very low	High

Abbreviations: ALD, atomic layer deposition; CVD, chemical vapor deposition; LPCVD, low-pressure CVD; PECVD, plasma-enhanced CVD.

**Table 6:** Examples of reaction mechanisms and applications for ALD layers

Type of ALD	Temperature range	Viable layers	Precursors/reactants	Applications
Catalytic ALD	>32 °C with Lewis base catalyst^4^	Metal oxides (that is, TiO_2_, ZrO_2_)^4^	TiCl_4_, ZrCl_4_, H_2_O (Ref. 4)	High k-dielectric layers, protective layers, anti-reflective layers, and so on^4^
Al_2_O_3_, ALD	30–300 °C	Al_2_O_3_, metal oxides^19^	Trimethyl aluminum, TiCl_4_, H_2_O, Ti(OiPr)_4_, (Metal)(Et)_2_ (Ref. 4)	Dielectric layers, insulating layers, and so on, solar cell surface passivation^18^
Metal ALD using thermal chemistry	175–400 °C (Ref. 19)	Metal fluorides, organometallics, catalytic metals^19^	M(C_5_H_5_)_2_, (CH_3_C_5_H_4_)M(CH_3_)_3_,Cu(thd)_2_, Pd(hfac)_2_, Ni(acac)_2_, H_2_ (Ref. 20)	Conductive pathways, catalytic surfaces, MOS devices^19^
ALD on polymers	25–100 °C (Ref. 4)	Al_2_O_3_, ZnO, TiO_2_, and metal oxides on for example, PET, PEN polyimide for flexible solar cells and displays^14^ and on textile^15^	Al(CH_3_)_3_, Ti(OiPr)_4_, (C_2_H_5_)_2_Zn, / H_2_O (Ref. 18)	Polymer surface functionalization and modification, creation of composites, diffusion barriers, and so on^4^
ALD on particles	25–100 °C for polymer particles, 100–400 °C for metal/alloy particles^4^	BN, ZrO_2_, CNTs, polymer particles	Various gases: use of rotary fluid bed reactor is highly important to ensure fluidation of particles^4,22^	Deposition of protective and insulating coatings, optical and mechanical property modification, formation of composite structures, conductive media
Plasma- or radical-enhanced ALD for single- and multiple-element ALD conductors	450–800 °C (Ref. 4)	Pure metals (that is, Ta, Ti, Si, Ge, Ru, Pt)^20^, and metal nitrides (that is, TiN, TaN, and so on)^4,22,23^	Organometallics, MH_2_Cl_2_, terbutylimidotris(diethylamido)tantalum (TBTDET), bis(ethylcyclopentadienyl)ruthenium, NH_3_ (Ref. 4)	DRAM structures, MOSFET and semiconductor devices, capacitors^19^ Cu- and Li-diffusion barrier layers^15,21^

Abbreviation: ALD, atomic layer deposition; MOS, metal–oxide–semiconductor. PET, Polyethylene terephthalate; PEN, Polyethylene naphthalate.

**Table 7:** Brief comparison of metals, ceramics and polymers for implantable applications^94^

Properties	Metal	Ceramic	Polymer
Biocompatibility	Good for limited metals	Good for bio-ceramics	Good for many polymers
Hermiticity	Good	Medium	Mostly poor
Degree of outgassing	Low	Low	Mostly high
Mechanical flexibility	Poor	Poor	Good
Reliability	Good	Good	Mostly poor
Optical transparency	Poor	Mostly poor	Good (for some)
RF transparency	Poor	Good	Good
Ease of processing	Difficult	Difficult	Good
Cost	High	High	Low
Relative weight	Heavy	Medium	Light
